# Understanding the Joule-heating behaviours of electrically-heatable carbon-nanotube aerogels†

**DOI:** 10.1039/d0na01002b

**Published:** 2020-12-28

**Authors:** Dong Xia, Heng Li, Peng Huang

**Affiliations:** School of Chemistry, University of Leeds Leeds LS2 9JT UK cmdx@leeds.ac.uk; Key Laboratory of Estuarine Ecological Security and Environmental Health, Tan Kah Kee College, Xiamen University 363105 Zhangzhou China; School of Engineering and Physical Sciences, Heriot-Watt University Edinburgh EH14 4AS UK p.huang@hw.ac.uk

## Abstract

Cylindrical monolithic, electrically-heatable reduced carbon nanotube (rCNT) aerogels were synthesized to exploit their Joule-heating behaviours using two different arrangements (*i.e.* the top–bottom arrangement and the side–side arrangement) under different compressive strains. The top–bottom arrangement results in faster heating kinetics (up to 286 K min^−1^), higher heating efficiency (up to 80 °C W^−1^), a more uniform temperature distribution profile and higher electro-thermal conversion efficiency than the side–side arrangement. This study provides fundamental perspectives for exploiting the Joule-heating behaviours of geometrically complex nanocarbon aerogels, and thus will have important implications in strain-sensitive materials.

## Abstract 2

Electrically-heatable carbon-nanotube aerogels under Joule-heating using the side–side arrangement and the top–bottom arrangement displaying significantly different heating behaviours.

## Graphical abstract

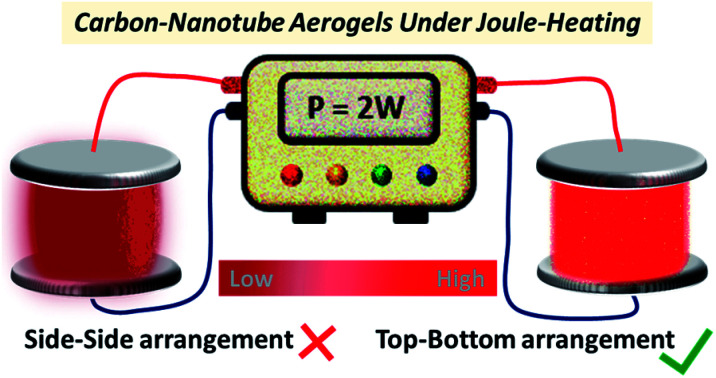

Nanocarbon aerogels, *e.g.* graphene aerogels^1^ and carbon nanotube aerogels,^2^ are three-dimensional (3D) monolithic, bulky materials. Their prominent features,^3–5^ such as a high surface area (up to 1230 m^2^ g^−1^),^6^ flexible mechanical properties (mechanical robustness of up to 96 kPa),^7^ diverse internal microstructures (*e.g.* mesopores, and macropores),^8,9^ high porosities, customisable shapes (*e.g.* cylinders, cubes, bricks, and five-pointed stars),^10–12^ ultra-low densities and high conductivities (electrical conductivity of up to 1000 S m^−1^ and thermal conductivity of up to 88.5 W m K^−1^),^13,14^ have enabled extensive applications in a wide variety of fields, including sensors,^15^ heaters,^16^ supporting frameworks,^17^ sorbents,^18^ electronics,^19^ catalysts,^20^ solar evaporators,^21^*etc*. In particular, the electrical and thermal conductivities of nanocarbon aerogels have been widely exploited due to the unique character of direct electrical heating of the graphitic support (Joule-heating),^16,22,23^ coupled with their highly customisable shape to fit between electrodes to execute energy-efficient Joule-heating studies.^24^

Reduced graphene oxide (rGO) aerogels have been found to exhibit ultrafast heating and cooling kinetics *via* Joule-heating (10 K s^−1^), exceptional cycling stability and repeatability, and a uniform temperature distribution across the structure.^16^ Taking advantage of the support framework, various inorganic functional nanoparticles (*i.e.* metallic nanoparticles, metal oxides, other 2D materials, and clays) can be incorporated onto the porous structure at high loadings to enhance the performance of the aerogel (*e.g.* sensors, and energy storage), as well as to facilitate completely new industrial applications (environmental remediation, heterogeneous catalysis, *etc.*). The ultrafast heating/cooling kinetics of the aerogel allows for energy-efficient recovery of the exhausted nanoparticles at a very low energy cost *via* direct local electrical heating, compared with traditional external oven heating *via* radiation.^25^ For boron nitride (BN) embedded reduced carbon nanotube (rCNT) aerogels, the mechanically strong hybrid aerogel can be linearly heated up to 700 °C whilst maintaining its structural integrity.^26^ Although widely investigated for the electrical and thermal properties, some of the fundamental Joule-heating characteristics of the aerogel, such as performance under compression, connection arrangement with the electrodes, and the respective Joule-heating efficiency and temperature distribution profiles, are largely unknown and deserve thorough investigation.

In this work, a facile wet-chemical synthetic approach was utilised to fabricate electrically-heatable reduced carbon nanotube (rCNT) aerogels for Joule-heating studies (Fig. 1a). The aqueous mixture of oxidized CNTs (oCNT) and polymers (*i.e.* polyvinyl alcohol, and sucrose) were exfoliated using ultrasonication to form dispersions, followed by casting into a copper disk mould for nanocarbon assembling. During the freezing step, ice-crystals will grow unidirectionally from the bottom of the liquid mixture and separate the nanocarbon to form porous channels, leading to the formation of unidirectional microstructures in the oCNT hydrogels.^27^ Electrically-conducting 3D rCNT aerogels were obtained after freeze-drying and thermal reduction (Fig. 1b),^16,28^ during which the oxygenic functional groups were eliminated to form the rCNT. The internal microchannels and diverse porous microstructures of the aerogel were confirmed by the SEM micrographs (Fig. 1c and d). The highly interconnected microstructure of the carbon nanotube provided numerous connected pathways for the movement of the electrical current, an essential prerequisite for executing Joule-heating studies. The random large pores distributed in the aerogel were beneficial for shape recovery after compression. Nitrogen adsorption measurements of the aerogel showed a surface area of 182 m^2^ g^−1^, in line with reported nanocarbon aerogels using the ice-templating method. A typical type-IV curve of the nitrogen adsorption/desorption isotherm confirmed the existence of large amounts of mesopores (Fig. 1e). The average pore size of the aerogel was around 50 nm (Fig. 1f), with a mesopore volume of 0.67 cm^3^ g^−1^, as calculated from the BJH pore diameter. The as-prepared rCNT aerogel exhibited characteristic graphitic (002) and (100) X-ray diffraction peaks of nanocarbon materials (ESI, Fig. S1†). Fig. 1g and h show that a light-emitting diode (LED) bulb can be lit up using a 3 V button battery when connected through the aerogel in both top–bottom (TB) and side–side (SS) arrangements, highlighting the electric conducting feature of the aerogel.^29^ A house-built Joule-heating setup with adjustable compression deformation was used for the studying of the impact of different arrangements (*i.e.* TB in Fig. 1i and SS in Fig. 1j) on the electrical and thermal properties. To avoid breakdown of the aerogel structure, the maximum compressive strain was controlled at *ω* = 20% in this study. A thermocouple was inserted into the centre of the aerogel equidistant from the electrodes to record the Joule-heating core temperature (Fig. 1k). When an electric current is introduced in the circuit, local aerogel resistive heating will be induced and the corresponding Joule-heating core temperature (*T*_core_, °C), voltage (*U*, V), current (*I*, A) and power (*P*, W) will be simultaneously recorded (see the ESI, Fig. S2–S4†).

**Fig. 1:**
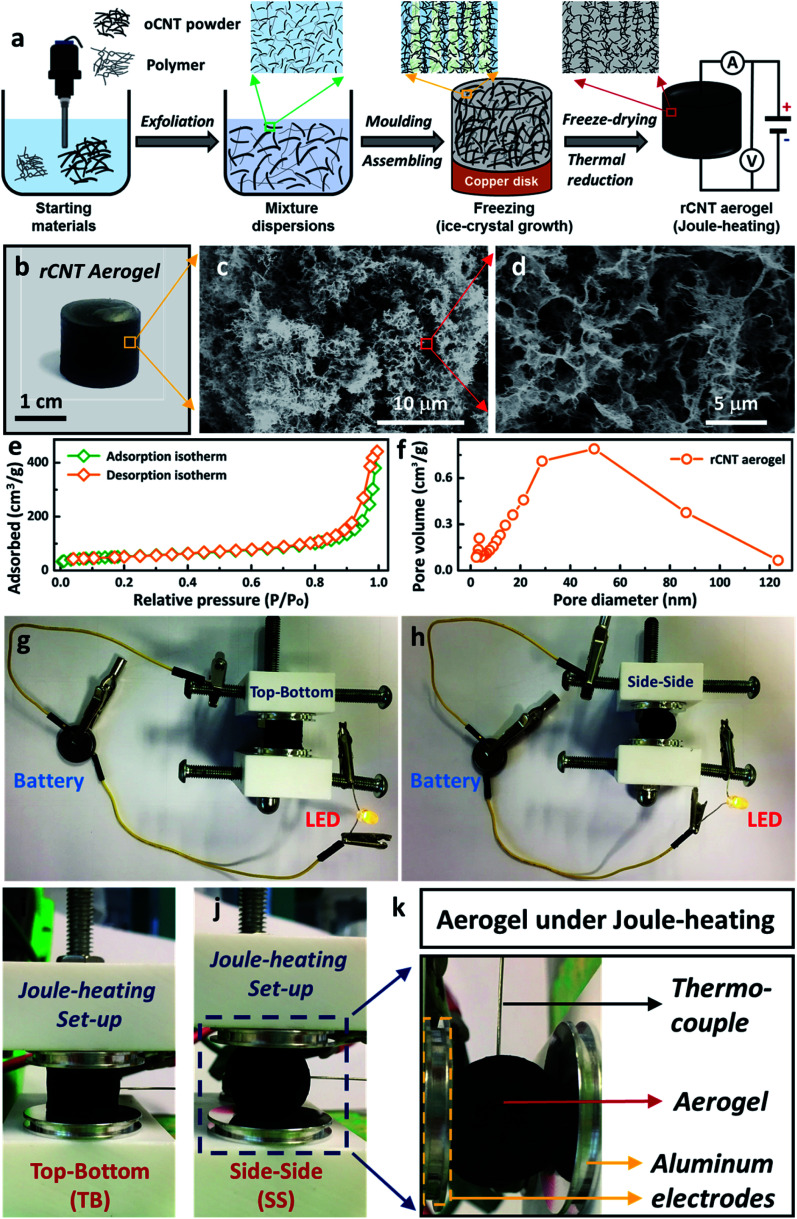
(a) Schematic description of the preparation of a rCNT aerogel. (b) Digital photo of a rCNT aerogel. (c and d) SEM images of the rCNT aerogel with different magnifications. (e) Nitrogen adsorption–desorption curves of the rCNT aerogel. (f) BJH pore size distribution of the rCNT aerogel. Lighting LED photos of rCNT aerogels: (g) top–bottom arrangement; (h) side–side arrangement. Digital photos of the rCNT aerogel under Joule-heating: (i) top–bottom heating; (j) side–side heating. (k) Detailed specification of an entire Joule-heating system.

The schematics of the aerogel under different compressions (*ω* = 5%, 10%, 20%) in both TB arrangement and SS arrangement are exhibited in Fig. 2a and b, respectively. All the *I*–*U* curves presented excellent linear relationships, indicating good electrical conductivity of the aerogel. The electrical resistance of the aerogel showed great dependence on the compressive strain (*i.e.* the more compressed the structure, the lower the electrical resistance), due to the formation of more compacted and interconnected attachments of the nanotubes under compressive strain (Fig. 2c and d).^30^ This feature is highly promising for important applications, such as stimulus-responsive graphene hybrids and pressure sensors. The decrease in electrical resistance under higher compressive strain also leads to lower core temperatures of the aerogel using the same Joule-heating setup (see the ESI, Fig. S5†). The voltage is significantly lower for the TB arrangement, decreasing from 4.7 V (*ω* = 5%) to 2.3 V (*ω* = 20%), than that of the SS arrangement at the same electrical current. This may be attributed to the different flow directions of the electric current between the electrodes, *i.e.* parallel to the unidirectional microstructure of the aerogel for the TB arrangement (Fig. 2g) and perpendicular for the SS arrangement (Fig. 2h). The electrical resistance and conductivity (*σ*, S m^−1^) variations under different compressions are shown in Fig. 2e, in which the electrical resistance decreased by around 50% in both arrangements. Consequently, the electrical conductivity has increased by 30% for the TB arrangement and 15% for the SS arrangement when the aerogel is compressed by 20%. Fig. 2f shows that the density of the aerogel gradually increased with the increase of the compressive strain, due to the reduction in volume. A density change of up to 30% has been observed for both TB and SS arrangements. The change in density has been found to impact significantly the thermal conductivity of aerogel materials.^31^ For both TB and SS arrangements, the voltage in the circuit increases with increasing power input, which follows well with Joule’s law (Fig. 2i and j).

**Fig. 2:**
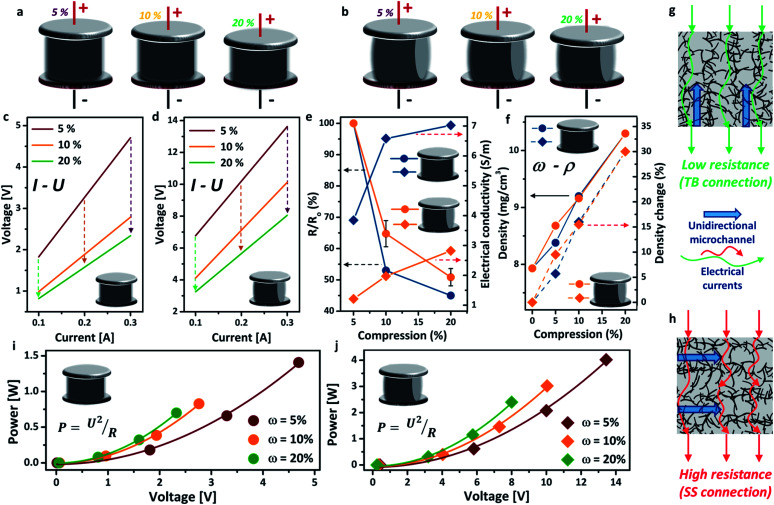
Schematic description of the different compressions of the rCNT aerogel for Joule-heating: (a) TB arrangement; (b) SS arrangement. *I*–*U* curves of the rCNT aerogel with different degrees of compression: (c) TB arrangement; (d) SS arrangement. (e) Compression *versus* resistance/electrical conductivity of the rCNT aerogel. (f) Compression *versus* density of the rCNT aerogel. Schematics of electrical currents through the TB arrangement (g) and the SS arrangement (h). *U*–*P* curves under different compressions: (i) TB arrangement; (j) SS arrangement.

The power–temperature dependence (*P*–*T*) of the aerogel under different compressive strains is shown in Fig. 3a and b. Both TB and SS arrangements exhibit good linear *P*–*T* relationships, with correlation coefficients greater than 0.997. These calibration curves demonstrate that the aerogel can be heated up to a relatively high temperature with very low energy consumption. The core temperature of the aerogel can also be precisely controlled by simply tuning the power input. For the TB arrangement, the aerogel showed a heating efficiency (defined by temperature over power,^16^ d*T*/d*P*) of 80.0 °C W^−1^ (*ω* = 20%), 33% higher than that of the SS arrangement under the same compressive strain. Much higher core temperatures were also achieved for the TB arrangement at the same power input, owing to its higher electrical conductivity. Interestingly, the temperature further increased when greater compressive strain was applied to the aerogel without changing the power input, which exhibits great potential as an energy-efficient heater.^32^ The thermal stability of the aerogel was tested by repeated heating and cooling at a fixed power input of 2 W, as presented in Fig. 3c and d. No decrease in Joule-heating performance was observed for both TB and SS arrangements after up to 10 cycles, corroborating the good thermal stability of the aerogel. The voltage curves suggest that the SS arrangement is more suitable for applications where a high voltage and low temperature are desirable, such as Li metal batteries^33^ and electrochemical capacitors.^34^ The heating rate of the aerogel reached 286 K min^−1^ for the TB arrangement and slightly slower for the SS arrangement. The cooling rates for both connection arrangements were similar, at around 400 K min^−1^ (Fig. 3e and f), mainly accredited to the high thermal conductivity and highly porous structure of the aerogel.

**Fig. 3:**
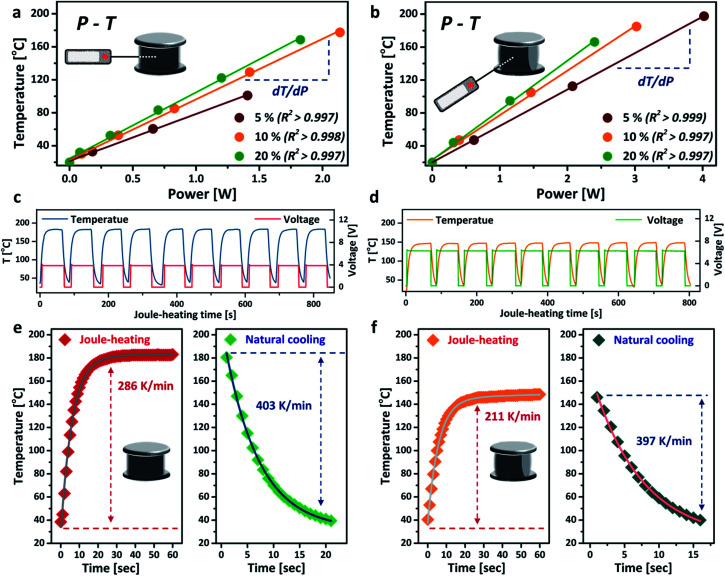
Joule-heating performance of rCNT aerogels under different compressions: (a) TB arrangement; (b) SS arrangement. Joule-heating cycles (core temperature, voltage) at power input 2 W: (c) TB arrangement, *ω* = 20%; (d) SS arrangement, *ω* = 20%. Heating/cooling kinetics of rCNT aerogels at power input 2 W. (e) TB arrangement, *ω* = 20%; (f) SS arrangement, *ω* = 20%.

To further investigate the impact of thermal conductivity on the cooling rates of the aerogel, a temperature gradient fitting method was employed. The temperature at a known distance from the centre, *r*, was recorded by using a thermocouple (Fig. 4a and b), and the collected data were fitted using a quadratic function (*T* = *T*_core_ − *C*_rad_ × *r*^2^, here *T*_core_ is the core temperature measured at 2 W, and *T* represents the temperature at a known distance from the centre, *C*_rad_ is the relevant quadratic fitting parameters). The thermal conductivity (*κ*, W m^−1^ K^−1^) was calculated from the equation *κ* = *q*/(4 × *C*_rad_), here *q* is the power density.^16,35^ The thermal conductivity of the aerogel for the TB arrangement, increased by 37% when compressed from 10% to 20% (Table S1, ESI†), due to the formation of a more interconnected nanostructure which lowers the resistance for phonon transport and facilitates heat transfer.^31,36^ However, the thermal conductivity of the aerogel remained almost constant at ∼0.117 W m^−1^ K^−1^ when using the SS arrangement. The similar thermal conductivities of the aerogel using both TB and SS arrangements under 20% compressive strain agreed with the relatively close cooling rates observed in Fig. 3e and f. Thermal images of the aerogel showed that the core temperature rises proportionally with the increase of the electrical current (Fig. 4c). At the same power input, the temperature distribution profiles were significantly different between the TB arrangement and the SS arrangement. The TB arrangement displayed uniform heating in the horizontal direction (red line in Fig. 4d, parallel to the aluminium plates), with very small temperature variation from the centre to the edge (Fig. 4f). However, a significant temperature drop was observed for the SS arrangement. In the vertical direction (perpendicular to the aluminium plates), the temperature gradient variations were great for both TB and SS arrangements, possibly due to the aluminium electrodes acting as the heat sink, which leads to substantial heat loss (Fig. 4g and i).^16^ These results demonstrate that the connection arrangement and compressive strain significantly impact the Joule-heating behaviours of nanocarbon aerogels.

**Fig. 4:**
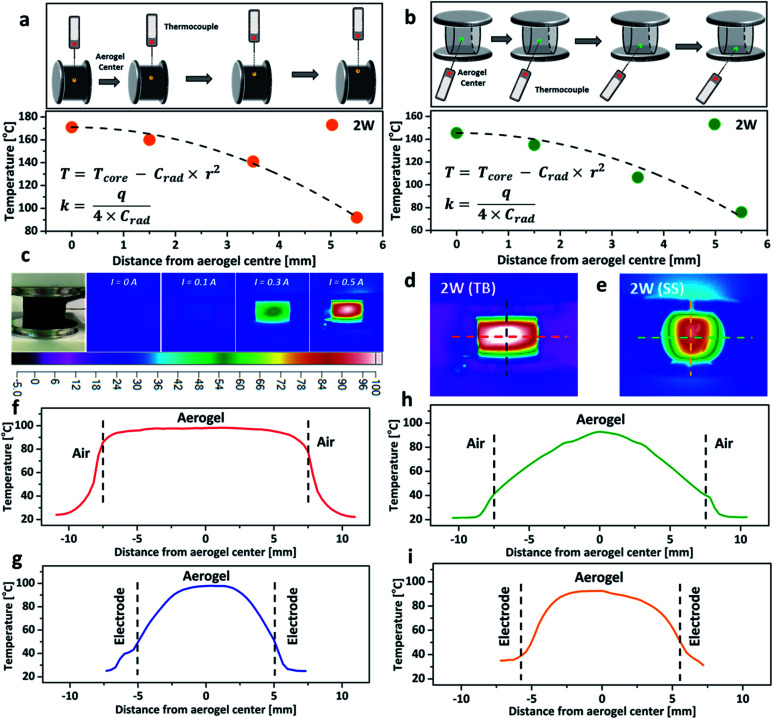
Schematic representation of the thermal temperature gradient fitting method of the rCNT aerogel and the correspondingly measured data (a) TB arrangement, *ω* = 10%; (b) SS arrangement, *ω* = 20%. (c) Thermal images of the rCNT aerogel at different electrical currents (TB arrangement, *ω* = 20%). Thermal images of the rCNT aerogel in the TB arrangement (d) and SS arrangement (e) with a *ω* = 20% at 2 W power input. (f and g) Transverse line and perpendicular line temperature distributions of the rCNT aerogel from (d). (h and i) Transverse line and perpendicular line temperature distributions of the rCNT aerogel from (e).

## Conclusions

Cylindrical monolithic rCNT aerogels were synthesised *via* a facile wet-chemical approach and the Joule-heating behaviours of the aerogels were investigated by employing two distinct arrangements (*e.g.* the top–bottom arrangement and the side–side arrangement) under different compressive strains in a house-built Joule-heating set-up. The results show that the Joule-heating efficiency of the aerogel increases with the rise of compressive strain, due to the formation of a more interconnected nanocarbon structure. The top–bottom arrangement exhibits higher Joule-heating efficiency, a faster heating rate, a more uniform temperature distribution, and better electrothermal energy conversion efficiency compared with the side–side arrangement. These differences in Joule heating characteristics are directly related to the anisotropic structure of the aerogel. Parallel movements of the electrical current along the unidirectional microstructure leads to more efficient electro-thermal conversion and less temperature gradient variation. This study provides a new perspective for optimizing the electro-thermal efficiencies of different types of nanocarbon aerogels, including graphene, graphene oxide, graphene nanofibers and carbon nanofiber aerogels, by exploiting the connection arrangements of the aerogel structure. More generally, this study highlights the importance of controlling the shape and geometry of the aerogel (*e.g.* cubes, cuboids, and cylinders) in applications that are based on Joule-heating, such as ultrafast and energy-efficient heaters, temperature swing sorption, temperature-dependent catalysis and stimulus-responsive graphene hybrids.

## Conflicts of interest

There are no conflicts to declare.

## Supplementary Material

NA-003-D0NA01002B-s001
