# Supplementary material for: Understanding the Joule-heating behaviours of electrically-heatable carbon-nanotube aerogels
Source: Nanoscale Adv. 2020 Dec 28;3(3):647–52. doi: 10.1039/d0na01002b (PMC9417603; doi:10.1039/d0na01002b)
Supplement: NA-003-D0NA01002B-s001 [file NA-003-D0NA01002B-s001.pdf]

Electronic Supplementary Material (ESI) for Nanoscale Advances.
This journal is © The Royal Society of Chemistry 2020

Supplementary Information

Understanding the Joule-Heating Behaviours of Electrically-Heatable Carbon-Nanotube Aerogels

Dong Xia*[a], Heng Li[b] and Peng Huang*[c]

[a]School of Chemistry, University of Leeds, Leeds, LS2 9JT, UK

[b]Key Laboratory of Estuarine Ecological Security and Environmental Health, Tan Kah Kee College, Xiamen University, 363105, Zhangzhou, China

[c]School of Engineering and Physical Sciences, Heriot-Watt University, Edinburgh, EH14 4AS, UK

*Corresponding author: cmdx@leeds.ac.uk; P.Huang@hw.ac.uk

Experimental section

Materials: Carboxylic-acid-functionalised multi-walled carbon nanotube (oCNT), sucrose and polyvinyl alcohol (PVA) were purchased from Sigma-Aldrich. HPLC water and toluene were supplied by Fisher Scientific UK. All chemicals were used as received.

Synthesis of reduced carbon nanotube (rCNT) aerogels: A polymer-assisted wet chemical synthetic method was used to fabricate the rCNT aerogels in this work. A mixture of 37.5 mg PVA and 37.5 mg sucrose were dissolved in 10 mL HPLC water in an oven. Then, 75 mg of oCNT powder was added into the mixture and tip-sonicated (model HD2200, BANDELIN electronic GmbH & Co. KG) four times to ensure good nanotube exfoliation (each sonication duration lasts for 5 min, with 30% of ultrasound power). After each sonication, the oCNT/PVA/sucrose mixture was vigorously agitated (3000 rpm/min) for 3 min using a vortex mixer to uniformly mix the dispersion. Into which, 2.5 mL toluene was added and vigorously agitated for another 5 min. Afterwards, 3.5 mL of the mixture was cast into a bespoke PTFE cylindrical mould, followed by freezing in liquid nitrogen for 10 min and freeze-drying for 48 hours. The resultant aerogels were thermally reduced at 1000 ^oC for 2 h in H_2/N_2 (5% H_2) atmosphere with a heating rate of 5 $^oC/min$ (Carbolite Gero Limited). The thermally reduced samples were designated as rCNT aerogels.

Joule-heating measurements: Joule-heating measurements of the prepared rCNT aerogels were carried out in a house-built Joule-heating setup. The aerogel was placed between the aluminium sample holders through both the top-bottom arrangement and the side-side arrangement to

record the Joule-heating parameters. Before the measurements, all the rCNT aerogels were preconditioned at a Joule-heating core temperature of 200 ^{o}C for 20 min to remove impurities (e.g. adsorbed water and volatiles). This step is essential for achieving stable and repeatable Joule-heating measurements in the following experiments. The stepwise Joule-heating measurements were performed by proportionally increasing the electrical current and held for 2 min at each step. The Joule-heating-induced temperature (measured by a thermocouple, K grounded tip insulated probe with a thermocouple data logger, RS Components Ltd) and voltage in both connection arrangements were recorded during the experiments. For the electrical-thermal stability test, the aerogel was repeatedly heated and cooled for at least 10 times with a power input of 2 W. Thermal conductivity of the aerogel calculated according to a previously reported radial temperature gradient fitting method.[7]

Materials characterisation: X-ray diffraction (XRD) pattern of the rCNT aerogel was collected on a Bruker D2 Phaser Diffractometer. Scanning electron microscopic (SEM) analysis was conducted on a Nova NanoSEM 450 with an accelerating voltage of 3 kV. The aerogel samples were fixed onto alumina stubs using conductive carbon adhesive tapes. Thermal images were taken using a Fluke TiR1 thermal camera. The obtained data were analysed using the Fluke Connect software. Brunauer-Emmett-Teller (BET) surface area of the aerogel was measured using a Micromeritics TriStar 3000 instrument. The samples were degassed under nitrogen atmosphere at 110 ^{o}C for 3 h before analysis. The nitrogen adsorption and desorption isotherms were measured at 77 K. The pore size distribution of the aerogel was calculated from the desorption isotherm using the Barrett-Joyner-Halenda (BJH) method.

Results and discussion

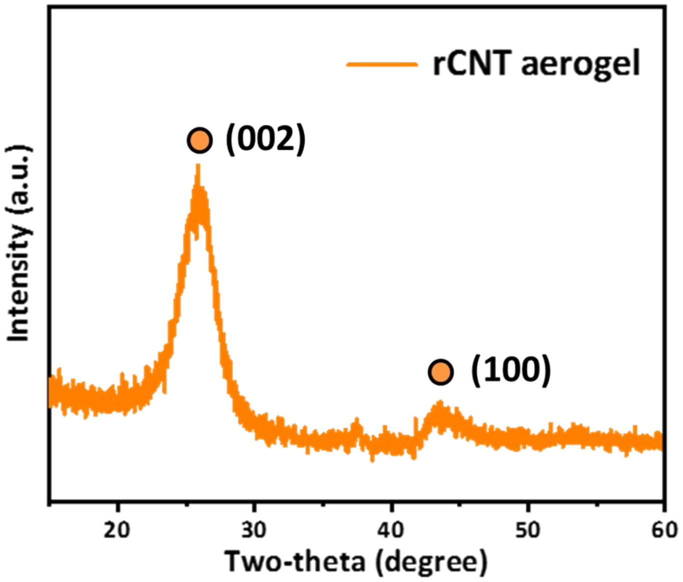

Fig. S1 XRD pattern of the reduced carbon nanotube aerogel.

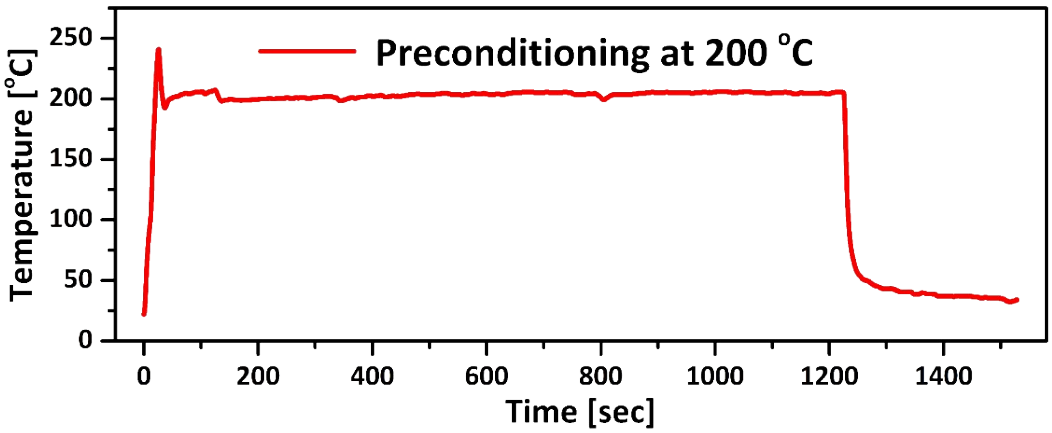

Fig. S2 Preconditioning at a temperature of 200 ^{o}C to stabilise the aerogel for the subsequent Joule-heating measurements.

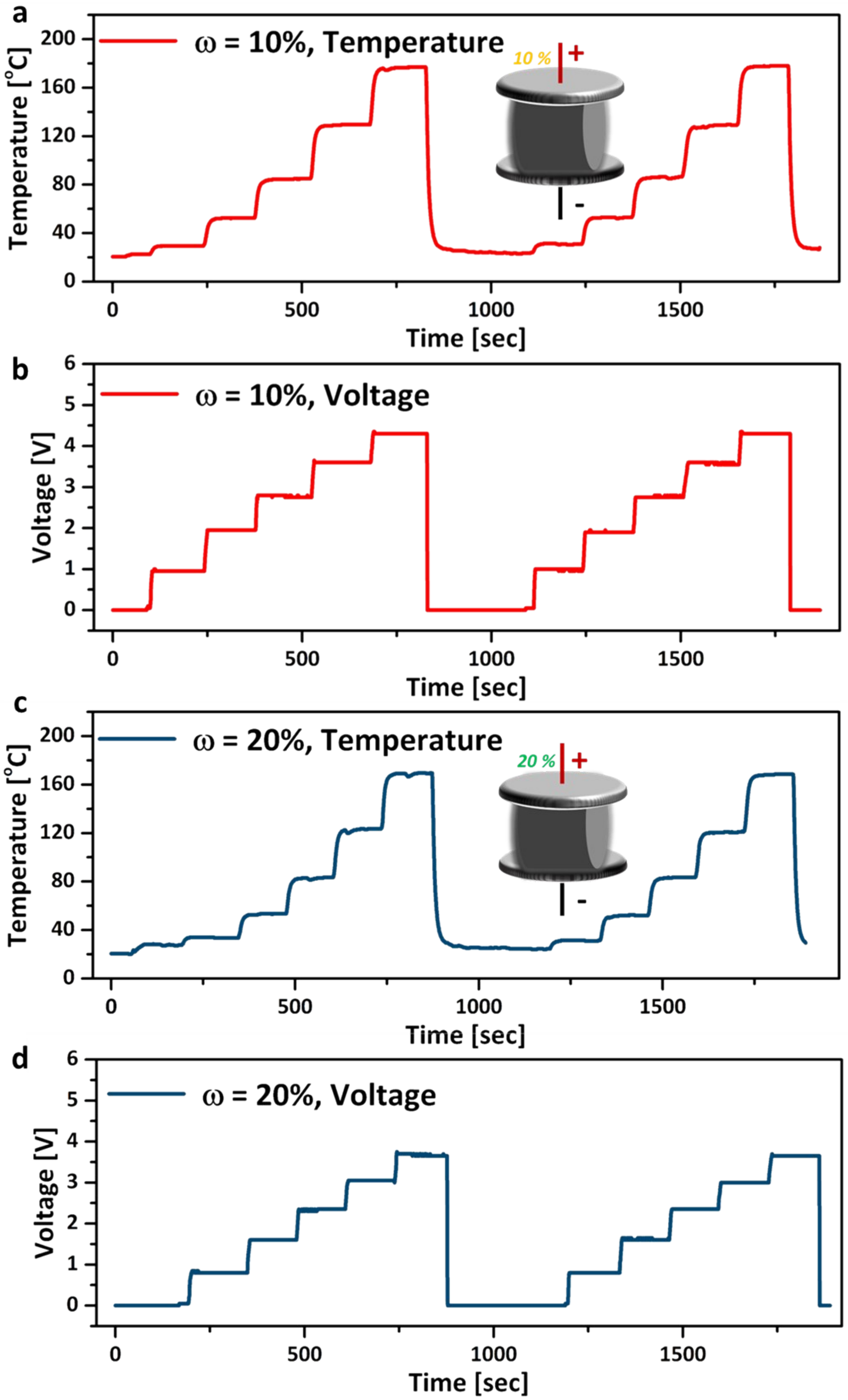

Fig. S3 Stepwise Joule-heating temperature and voltage curves of the aerogel using top-bottom arrangement: temperature (a) and voltage (b) under 10% compression; temperature (c) and voltage (d) under 20% compression.

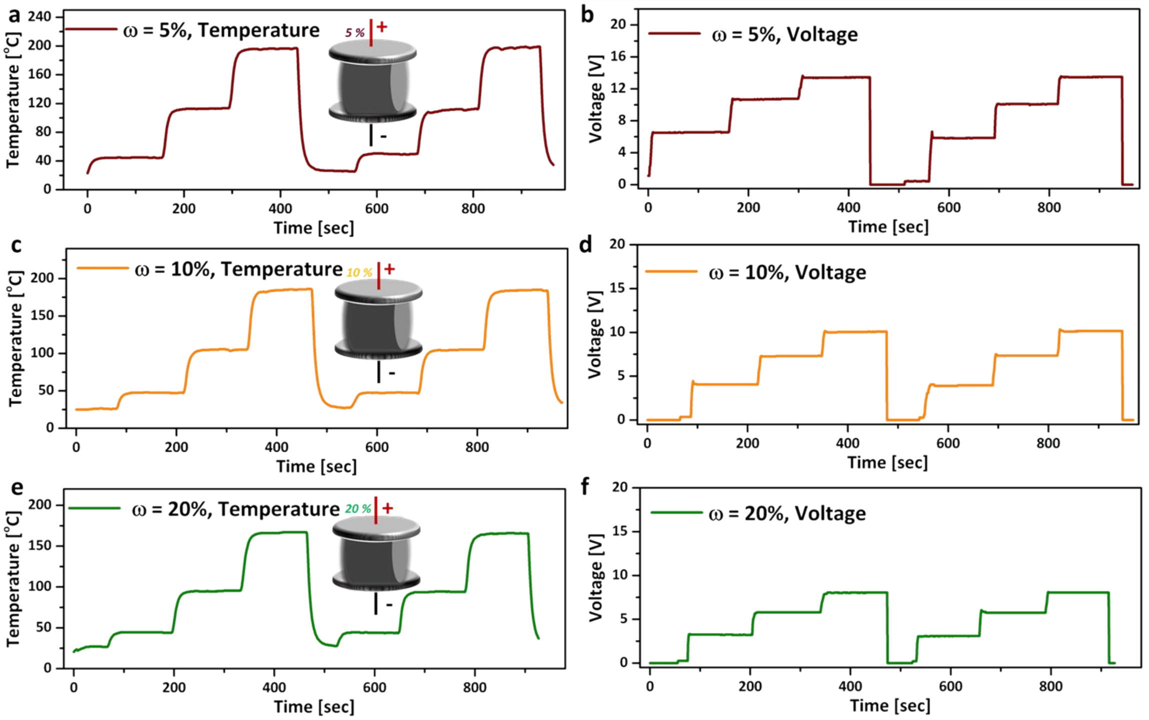

Fig. S4 Stepwise Joule-heating temperature and voltage curves of the aerogel using side-side arrangement: temperature (a) and voltage (b) under 5% compression; temperature (c) and voltage (d) under 10% compression; temperature (e) and voltage (f) under 20% compression.

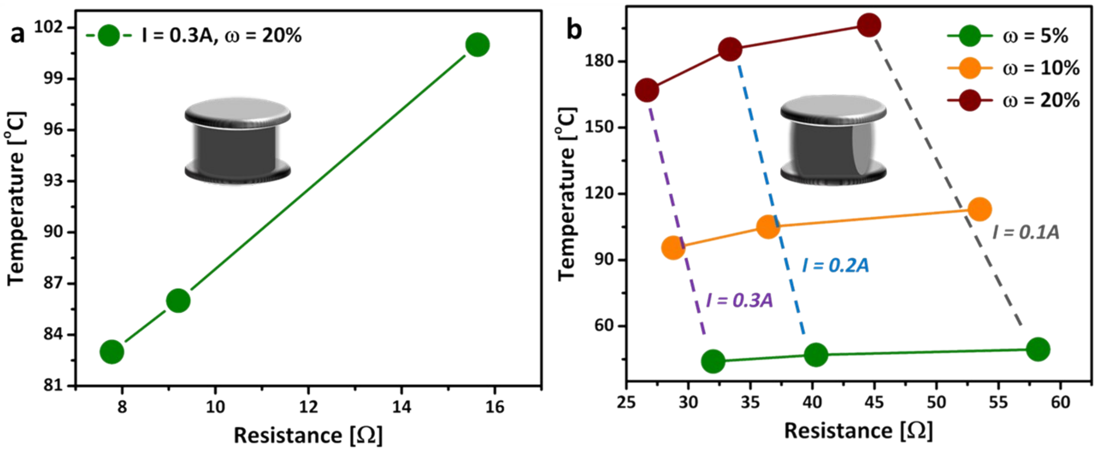

Fig. S5 Resistance-temperature curves of the aerogel under different compressions: (a) top-bottom arrangement; (b) side-side arrangement.

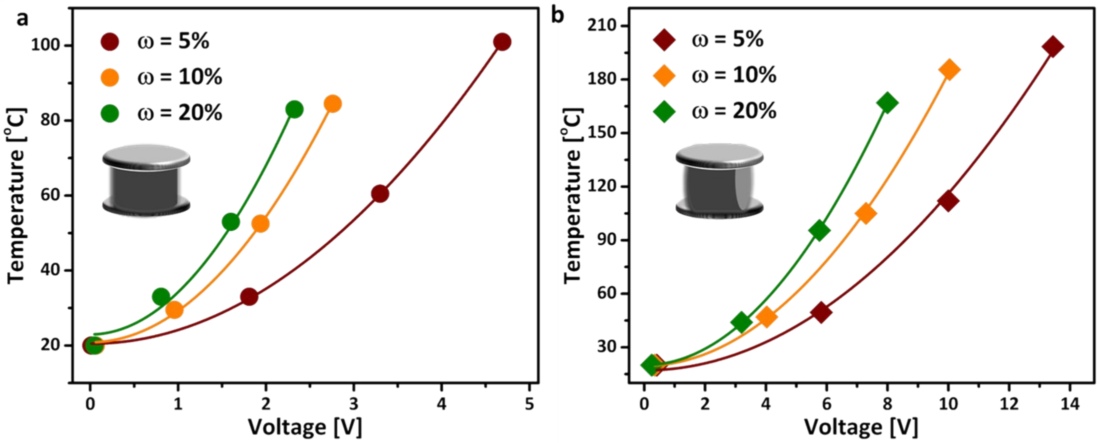

Fig. S6 Voltage-temperature curves of the aerogel under different compressions: (a) top-bottom arrangement; (b) side-side arrangement.

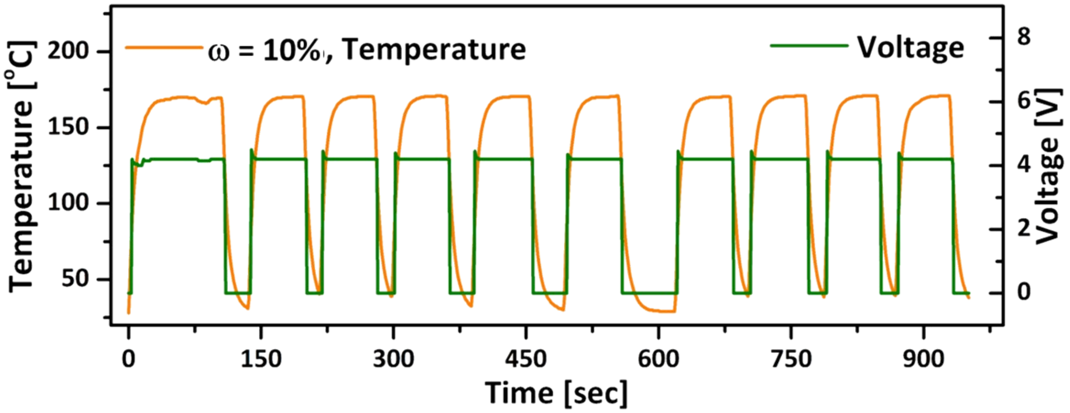

Fig. S7 Joule-heating cycles (core temperature, voltage) at a power input of 2W using top-bottom arrangement under 20% compression.

Table S1 Joule-heating parameters of the aerogels using top-bottom arrangement and side-side arrangement.

Connection arrangements	Compression (%)	Volume (cm^3)	q (10^6 W·m^{-3})	T_{core} (°C)	C_{rad} (10^5 K·m^{-2})	k (W·m^{-1}·K^{-1})
Top-Bottom	10	9.2	1.03	171	3.19	0.081
Top-Bottom	20	10.3	1.16	184	2.60	0.111
Side-Side	5	8.7	0.99	118	2.11	0.117
Side-Side	10	9.2	1.02	136	2.25	0.114
Side-Side	20	10.3	1.34	145	2.43	0.117

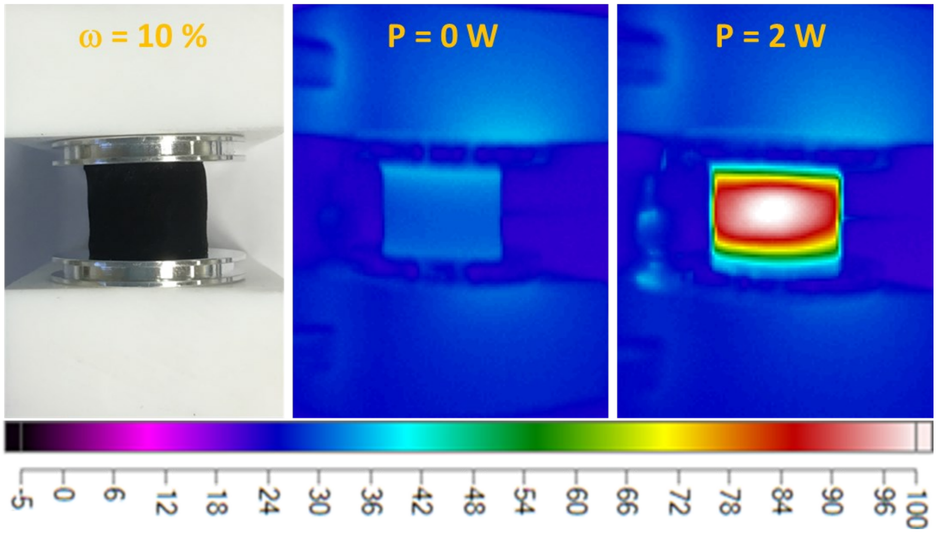

Fig. S8 A digital photo and thermal images of the aerogel under 10% compression using top-bottom arrangement.

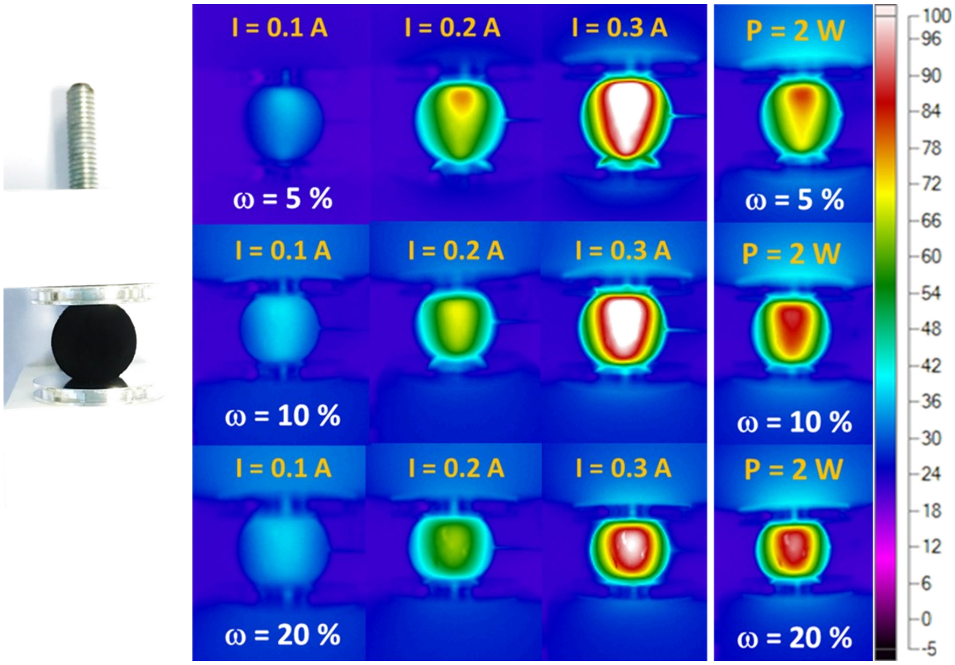

Fig. S9 A digital photo and thermal images of the aerogel under different compressions using side-side arrangement.
